# Evidence for the Association of a Deleted Variant in the 5′-Flanking Region of the Chicken *serotonin transporter (5-HTT)* Gene with a Temporary Increase in Feed Intake and Growth Rate

**DOI:** 10.3390/ani6100063

**Published:** 2016-10-14

**Authors:** Joergen B. Kjaer, Loc Phi-van

**Affiliations:** Institute of Animal Welfare and Animal Husbandry, Friedrich-Loeffler-Institut, Dörnbergstraße 25/27, Celle 29223, Germany; joergen.kjaer@fli.de

**Keywords:** appetite, feed intake, body weight, chicken *5*′-*HTT*, ghrelin

## Abstract

**Simple Summary:**

The serotonergic system has been shown to be implicated in the regulation of mood and feeding behavior. Previous studies have identified a polymorphism in the 5′-flanking region of the *serotonin transporter* (*5*-*HTT*) gene of Lohmann Brown (LB) laying hens. In this study, the impact of three genotypes (W/W, W/D, and D/D) at the *5*-*HTT* gene on feed intake and body weight was investigated. After hatching, hens of the three genotypes were kept under ad libitum feeding conditions, and their feed intake and body weight were recorded weekly. From 5 weeks of age, D/D and W/D hens were significantly heavier than wild-type W/W hens. Further, it is found that D/D hens had a temporarily higher feed intake and growth rate than W/W hens between 4 and 7 weeks. We suggest that the increased body weight is due to a transiently increased appetite of D/D hens.

**Abstract:**

The serotonergic system has been shown to be implicated in the regulation of mood and feeding behavior. Previous studies have identified a polymorphism in the 5′-flanking region of the *serotonin transporter* (*5*-*HTT*) gene of Lohmann Brown (LB) laying hens. The deleted variant D was found to be associated with increased body weight. The objective of this study was to address whether the increased body weight may be due to an increased feed intake. After hatching, hens were kept under ad libitum feeding conditions, and their body weight and feed intake were weekly determined. From 5 weeks of age, the body weight of hens with the D/D and W/D genotypes was significantly greater than that of W/W carrying hens. Interestingly, we found that the feed intake of D/D carrying hens, relative to body weight, was transiently increased only between 4 and 7 weeks of age (*p* < 0.05), leading to a higher growth rate (*p* < 0.05), compared with that of W/W carrying hens. These results suggest that the presence of variant D may be correlated with a transiently increased appetite of D/D carrying hens.

## 1. Introduction

The nervous system regulates energy balance through behavioral, physiological, and metabolic processes. Several studies have provided increasing evidence indicating that neuronal serotonin (5-hydroxytryptamine, 5-HT) is involved in the processes of regulating food intake and body weight. Indirect 5-HT agonists such as fenfluramine and 1-(3-chlorophenyl) piperazine or the selective serotonin reuptake inhibitors fluoxetine and paroxetine, which increase synaptic 5-HT concentrations, have been shown to significantly reduce food intake and to induce body weight loss in both animals and humans [1,2,3,4,5]. Marked hypophagia and reduced body weight were also observed in rodents by direct injection of 5-HT into the paraventricular nucleus (PVN) of the hypothalamus [6] or by peritoneal injection of its precursors, tryptophan and 5-hydroxytryptophan [7,8]. Conversely, inhibition of the 5-HT synthesis via the administration of p-chlorophenylalanine, which reduces synaptic 5-HT, results in increased food intake and body weight [9]. The role of 5-HT receptors was first indicated by pharmacological studies using selective 5-HT agonists and antagonists. These studies reveal that the 5-HT_2C_ receptor, which is most highly expressed in the hypothalamus [10], is responsible for the negative 5-HT effect on appetite and food intake [5]. Unsurprisingly, the recently approved selective 5-HT_2C_ agonist lorcaserin is a successful drug to reduce body weight in anti-obesity therapy in humans [11,12]. In line with these findings, solid evidence indicating the role of the 5-HT_2C_ receptor in the control of appetite and energy balance comes from studies using knockout mice, which have demonstrated that knockout mice deficient of the serotonin receptor 5-HT_2C_ develop chronic hyperphagia and late-onset obesity [13,14]. 

Previously, we reported a functional polymorphism of the 5′-flanking region of the chicken *5*-*HTT* gene of the Lohmann Brown (LB) layer line [15]. The polymorphism is generated by deletion of four nucleotides (5′-AATT-3′) and a closely spaced single nucleotide change (A → T). The presence of the variant D in the 5′-flanking region of the *5*-*HTT* gene results in an increase in 5-HTT expression. Additionally, hens carrying the deleted variant D exhibit increased body weight and locomotor activity compared with hens with the wild-type variant W. Therefore, the main aim of this study was to examine the increase in body weight in further detail. We asked the question of whether an increase in feed intake due to increased appetite may be the reason for the increased body weight of D- and W-carrying hens.

## 2. Materials and Methods

### 2.1. Animals

Chickens were generated as described previously [15]. For experiments of determining feed intake, after hatching, hens were sorted and partitioned according to genotype in W/W, W/D, and D/D genotype groups. Because D/D and W/D hens exhibited an increase in body weight gain earlier than D/D and W/D cocks [15], we used only hens for this study. In order to obtain enough data for statistical evaluation, hens of each genotype were divided into 6 independent groups (15 hens each). They were maintained in a floor system on a 10-h dark-14-h light cycle at 18 °C and had ad libitum access to feed and water. Care and use of animals for the purpose in this study followed the guidelines of the European Communities Council Directive of 22 September 2010 (2010/63/EU) and the German Animal Protection Law.

### 2.2. Body Weight and Feed Intake

Body weight of individual hens was measured weekly. Feed consumption was recorded weekly for a time period of 11 weeks as follows. Feed weighed previously was provided separately to all groups of hens with different genotypes, leftovers collected were weighed on the following week, and the difference was calculated as mean weekly feed intake per hen and then normalized to mean body weight as weekly group average feed intake per kg of body weight.

### 2.3. Determination of Ghrelin

Plasma ghrelin levels were determined at 6 weeks of age using an ELISA kit from Cusabio Biotech (Wuhan, China) according to the instructions of the manufacturer.

### 2.4. Statistical Analysis

Body weights and feed intake were normally distributed and analyzed for each age according to the three genotypes using a one-way ANOVA followed by Tukey’s HSD post hoc test. The differences were considered statistically significant if the *p*-values were smaller than or equal to 0.05. Statistical analyses were performed using the VassarStats calculator (Vassar College, Poughkeepsie, NY, USA). The average growth rate (g/day) of hens was calculated post hoc for the period before, during, and after significant genotype differences in feed intake (see below), i.e., Period A for Weeks 1 to 4, Period B for Weeks 5 to 7, and Period C for Weeks 8 to 11. Growth rate was tested for effect of genotype within each of these periods using the GLM procedure in SAS (Statistical Analysis Systems Inc., Cary, NC, USA) with the following model: log(growth rate) = Genotype*_i_* + e*_ij_*, where *i* = (W/W, W/D, D/D), and e*_ij_* is N(0,1). Back-transformed values are presented. Outliers (*n* = 11) were discarded when values were below 4 and over 15 g/day.

## 3. Results and Discussion

Table 1 presents the effect of the genotypes on the body weight of the hens. No significant difference in the mean body weight of hens with different genotypes was found during the first 4 weeks after hatching. From the age of 5 weeks, the mean body weight differed between genotypes with D/D- and W/D-carrying hens, having significantly greater body weight than W/W hens. The maximum difference in body weight between W/W and D/D hens was about 10%. These results raised the question of whether the feed intake of D/D and W/D hens may be higher than that of W/W hens. Table 2 (a) shows that the feed intake in relation to body weight was highest in the first week after hatching, and this was continuously decreasing over the following seven weeks. In line with the results presented in Table 1, the feed intake of D/D hens was significantly higher than that of W/W hens from 4 to 7 weeks of age. In this time period, the feed intake of W/D hens was between those of W/W and D/D hens, but did not significantly differ from any of the two groups. Afterwards, the feed intake of D/D and W/D hens was reduced to the feed intake level of W/W hens.

Based on the temporary significant genotype difference in feed intake between 4 and 7 weeks of age, average daily growth rate was calculated post hoc for the period before, during, and after these significant genotype differences, and results are presented in Table 2 (b). Only in the middle period, from 5 to 7 weeks of age, the growth rate was different between hens of the three genotypes. The post-hoc test revealed that the difference between D/D and W/W and between W/D and W/W were significant, whereas the difference between D/D and W/D was not significant. This shows that the higher feed intake in these weeks indeed resulted in a higher growth rate.

It is well known that ghrelin is an endogenous orexigenic factor in rodents, and that plasma ghrelin levels increase after fasting, which in turn decrease after feeding [16,17]. Therefore, we determined the plasma ghrelin levels in hens at 6 weeks of age. We found that the plasma ghrelin levels were not significantly different between hens of the three genotypes (data not shown). Thus, ghrelin seems not to function as an orexigenic factor in chickens, as also reported by several authors [18].

Our previous study demonstrated that hens of the D/D genotype were physically more active than hens of the other genotypes. The question as to why D/D hens were more active and had nevertheless higher body weight than W/W hens has been raised. A possible explanation for this observation was that they had ad libitum access to feed and water, and consumed more feed to compensate for the higher energy expenditure caused by increased locomotion activity. In addition, the energy surplus leading to the fast growth rate might result from the increased feed intake which is thought to be mediated by the higher *5-HTT* expression in D/D hens.

Little data exist so far indicating the role of 5-HT and *5-HTT* in the control of appetite or feed intake in chickens. These data have come almost exclusively from pharmacological studies performed with intraventricular injection of 5-HT or using fenfluramine and the selective 5-HTT inhibitor fluoxetine [19,20,21]. Recently, studies on the 5-HT effects after central injection revealed that the anorexigenic effect of 5-HT was not associated with central pathways controlling the appetite and feed intake, but was likely secondary to the increased deep rest and other behaviors including reduced feed pecking [22]. This issue warrants further study with hens of the three genotypes W/W, W/D, and D/D. However, consistent with the findings indicating the anorexigenic effect of 5-HT, our present data show for the first time that hens carrying D/D alleles, which are associated with higher *5-HTT* expression, exhibited a higher feed intake compared with those of the wild-type W/W genotype, but only for a limited time (from 4 to 7 weeks of age). This raised the question as to the nature of the *5-HTT* expression during this time period and whether it is correlated with the temporarily increased feed intake of D/D hens, which warrants further exploration. Perhaps, growth factors, such as growth hormone (GH) and insulin-like growth factor-I (IGF-I), might interact with 5-HT pathways to influence feed consumption and growth [23]. GH and IGF-I are necessary for the early growing phase of chickens. In fact, it has been shown that plasma IGF-I levels increase progressively from 0 to 4 weeks of age and remain elevated to 7 weeks of age, thereafter declining and reaching a basal level at 10 weeks of age [24,25]. 

## 4. Conclusions

In conclusion, results found in this study reveal that, compared with the W/W genotype, W/D and D/D genotypes were associated with increased body weight as previously observed. The increased body weight gain was caused by a temporary higher feed intake of hens between 4 and 7 weeks of age. To the best of our knowledge, the present results provide evidence for the first time that *5-HTT* gene variants are associated with changes in feed intake and body weight in chickens.

## Figures and Tables

**Table 1:** Body weight development of hens of different genotypes (mean ± SEM).

Age (Week)	W/W (*n* = 90)	W/D (*n* = 90)	D/D (*n* = 90)	*p*-Value
	Body Weight (g)			
1	62.1 ± 0.90	64.8 ± 0.71	64.1 ± 0.81	n.s.
2	105.4 ± 0.98	109.0 ± 1.27	109.2 ± 1.36	n.s.
3	161.6 ± 1.79	169.2 ± 2.09	167.3 ± 1.95	n.s.
4	231.6 ± 2.82	239.2 ± 3.35	236.7 ± 2.89	n.s.
5	308.3 ^b^ ± 4.18	319.2 ^a^ ± 4.46	322.4 ^a^ ± 4.01	<0.05
6	394.0 ^b^ ± 5.35	414.8 ^a^ ± 5.78	420.0 ^a^ ± 7.25	<0.05
7	485.2 ^b^ ± 6.62	517.2 ^a^ ± 6.73	527.3 ^a^ ± 6.99	<0.01
8	585.7 ^b^ ± 7.60	625.9 ^a^ ± 7.51	638.2 ^a^ ± 8.88	<0.01
9	658.0 ^b^ ± 8.34	707.2 ^a^ ± 9.08	725.1 ^a^ ± 9.56	<0.01
10	732.3 ^b^ ± 9.62	783.7 ^a^ ± 9.86	795.0 ^a^ ± 10.64	<0.01
11	818.2 ^b^ ± 10.38	877.9 ^a^ ± 11.14	883.6 ^a^ ± 11.57	<0.01

W: wild-type *5*-*HTT* genotype; D: variant *5*-*HTT* genotype with a 4 base deletion (AATT) and one base change (A → T) in the 5′-flanking region; n: number of hens of each genotype; ^a,b^ Means in a row with no common superscript letter differ significantly; n.s.: not significant.

**Table 2:** Feed intake and growth rate of hens. (a) Feed intake of hens of different genotypes (g per kg body weight per week) (mean ± SEM); (b) growth rate (g/day) for Period A (Weeks 1 to 4), Period B (Weeks 5 to 7), and Period C (Weeks 8 to 11) (lsmean ± SEM, *N* = 257).

**(a)**				
**Age (Week)**	**W/W (*n* = 6)**	**W/D (*n* = 6)**	**D/D (*n* = 6)**	***p*-Value**
0–1	1736.5 ± 71.23	1916.2 ± 59.24	1888.0 ± 54.05	n.s.
1–2	1691.7 ± 40.55	1688.8 ± 49.04	1676.8 ± 43.53	n.s.
2–3	1343.8 ± 26.60	1455.5 ± 42.27	1386.3 ± 32.11	n.s.
3–4	1139.9 ± 16.58	1237.7 ± 35.84	1183.2 ± 28.14	n.s.
4–5	919.1 ^b^ ± 21.05	975.0 ^a,b^ ± 31.39	1016.2 ^a^ ± 16.11	<0.05
5–6	833.9 ^b^ ± 15.92	888.0 ^a,b^ ± 26.29	904.5 ^a^ ± 13.68	<0.05
6–7	730.6 ^b^ ± 12.15	745.8 ^a,b^ ± 10.72	779.0 ^a^ ± 11.62	<0.05
7–8	678.2 ± 6.87	683.4 ± 3.78	689.9 ± 7.12	n.s.
8–9	565.0 ± 7.15	566.9 ± 9.85	583.4 ± 12.91	n.s.
9–10	595.5 ± 8.72	575.1 ± 4.57	577.1 ± 12.68	n.s.
10–11	596.5 ± 3.71	598.9 ± 8.93	596.2 ± 7.55	n.s.
**(b)**				
**Period ^1^**	**W/W**	**W/D**	**D/D**	***p*-Value**
A	6.13 ± 0.09	6.30 ± 0.09	6.20 ± 0.09	n.s.
B	8.50 ^b^ ± 0.15	9.44 ^a^ ± 0.15	9.78 ^a^ ± 0.15	<0.001
C	8.32 ± 0.17	8.74 ± 0.17	8.90 ± 0.17	n.s.

W: wild-type *5*-*HTT* genotype; D: variant *5*-*HTT* genotype with a 4 base deletion (AATT) and one base change (A → T) in the 5′-flanking region; *n*: number of independent groups (data points), each consisting of 15 hens; *N*: number of independent data points, each consisting of one individual hen; ^1^ The periods were defined post hoc as the period before, during, and after significant genotype differences in feed intake (see (a)); ^a,b^ Means and lsmeans in a row with no common superscript letter differ significantly (*p* < 0.05); n.s.: not significant.
